# Monitoring Information Flow on Coronavirus Disease 2019 (COVID-19)

**DOI:** 10.31138/mjr.31.3.243

**Published:** 2020-09-08

**Authors:** Armen Yuri Gasparyan, Olena Zimba, Durga Prasanna Misra, George D. Kitas

**Affiliations:** 1 Departments of Rheumatology and Research and Development, Dudley Group NHS Foundation Trust (Teaching Trust of the University of Birmingham, UK), Russells Hall Hospital, Dudley, West Midlands, United Kingdom,; 2 Department of Internal Medicine #2, Danylo Halytsky Lviv National Medical University, Lviv, Ukraine,; 3 Department of Clinical Immunology and Rheumatology, Sanjay Gandhi Postgraduate Institute of Medical Sciences, Lucknow, India; 4 Arthritis Research UK Epidemiology Unit, University of Manchester, Manchester, United Kingdom

**Keywords:** COVID-19, hydroxychloroquine, information, periodicals as topic, retractions, social media

## Abstract

The flow of information on Coronavirus Disease 2019 (COVID-19) is intensifying, requiring concerted efforts of all scholars. Peer-reviewed journals as established channels of scientific communications are struggling to keep up with unprecedented high submission rates. Preprint servers are becoming increasingly popular among researchers and authors who set priority over their ideas and research data by pre-publication archiving of their manuscripts on these professional platforms. Most published articles on COVID-19 are now archived by the PubMed Central repository and available for searches on LitCovid, which is a newly designed hub for specialist searches on the subject. Social media platforms are also gaining momentum as channels for rapid dissemination of COVID-19 information. Monitoring, evaluating and filtering information flow through the established and emerging scholarly platforms may improve the situation with the pandemic and save lives.

## INTRODUCTION

The coronavirus disease 2019 (COVID-19) pandemic has affected all aspects of human life, including healthcare, research and development, which are now preoccupied with fighting the disease.^1^ The growing number of COVID-19 cases and related deaths necessitates a balanced analysis of the news reports and research articles which are multiplying weekly. As of June 10, 2020, 50,632 COVID-19 articles, 3,506 related clinical trials protocols and reports, and 803 policy documents were retrievable on Dimensions.ai (https://covid-19.dimensions.ai/), which is one of the largest digital search and analytics platforms.

There are some promising initiatives to accumulate evidence and supply the World Health Organization and other global institutions with information that can be useful to doctors on the front lines combating COVID-19 and saving thousands of human lives (https://authors.bmj.com/policies/covid-19/).

Scholarly journals are operating in conditions of shortage or unavailability of peer reviewers due to the pandemic. The increased flow of submissions on the new coronavirus, however, necessitates mobilizing available resources and shortening turnaround times. An unpublished analysis of 529 articles in 14 influential journals with interest in virology and infectious diseases revealed substantial shortening of average turnaround times: from 117 days before to 60 days during this pandemic.^2^ The study suggests that both editorial assessment and peer review timelines in the medical journals have nearly halved, which might potentially affect the publication quality negatively.The unprecedented difficulties with sharing knowledge via established publishing channels bring numerous news outlets, online scholarly platforms, and popular social media to the fore. These public platforms allow posting of variable quality (non-peer-reviewed) materials to set priority over the user ideas, hypotheses, and scientific facts.

## EMERGING COVID-19 RESOURCES

**Figure 1.**
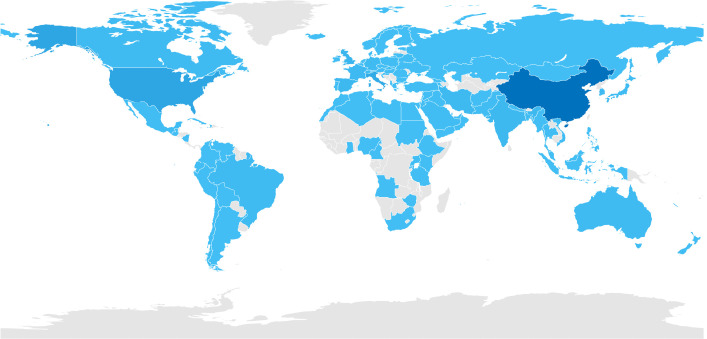
A screenshot of LitCovid homepage with countries mentioned in abstracts (as of June 10, 2020). No mentions relate to countries depicted in grey.

Some of the established scholarly resources are now accumulating COVID-19 documents on a daily basis. The established indexing services, particularly PubMed/MEDLINE and Scopus, which are often recommended for comprehensive coverage and systematic synthesis of evidence-based data, continue serving professional interests of all those seeking updates on COVID-19. Earlier this year, the US National Institutes of Health’s intramural research programme responded to the flooding of scientific information on the novel coronavirus and organized LitCovid, a PubMed-based literature hub and advanced search tool (https://www.ncbi.nlm.nih.gov/research/coronavirus/). LitCovid allows retrieving 35% more COVID-19-tagged articles than established search platforms.^3^ As of now, 20,404 PubMed-indexed articles are available for retrieval on LitCovid. The articles are categorized by topics related to various aspects of the disease, chemicals, target journals, article types, and countries mentioned in the abstracts. Hydroxychloroquine, chloroquine, remdesivir, lopinavir-ritonavir combination and tocilizumab are drugs commonly mentioned in the articles. The *BMJ*, *Journal of Medical Virology*, *The Lancet*, *The New England Journal of Medicine* and *Clinical Infectious Diseases* are the top five target journals. While China, the United States, Italy, the United Kingdom, and Spain are the top five countries frequently discussed in the articles, there are countries without any linked records in the hub (eg, Central Asian republics, except Kazakhstan) (Figure 1).

A number of ahead-of-print publications on COVID-19 are displayed on the databases’ search platforms without definite tagging with structured keywords, which might hinder their processing for systematic syntheses of evidence.

Numerous potentially valuable unpublished items and publications on COVID-19 that do not meet the criteria for indexing can be easily missed by authors if they search only through the established databases. As such, authors are advised to perform complementary searches on publishing platforms of top evidence-based journals which rapidly process and post influential COVID-19 articles ahead of print (eg, *The Lancet*, *BMJ*, *Nature*). Additionally, globally popular preprint servers, such as medRxiv.org and bioRxiv.org, are now publicly archiving non-reviewed opinion pieces and research reports, categorized as unpublished documents, which may have implications well before their publication in peer-reviewed journals. Both these popular preprint servers have already posted nearly 3,000 documents on COVID-19.^4^ Searching through the preprint servers and citing the archived reports may enrich future studies aiming at developing effective treatment and preventive measures during the pandemic. Readers and authors, however, are advised to use their judgement to distinguish testable hypotheses and verifiable facts from fiction and poor preprint reports as these may adversely affect patients with COVID-19.^4^ Many preprint reports are poorly edited and lack essential information about COVID-19 patient characteristics.^5^ As an example, flawed studies on hydroxychloroquine posted on preprint servers attracted numerous citations which might create a false impression of their approval by the global scholarly community.^5^ The authors who refer to the widely circulated preprints on hydroxychloroquine and antivirals are advised to recheck these items’ status since some have already been retracted.^6^

COVID-19 articles can be enriched by referring to daily statistics on its morbidity and mortality available on a specifically designed platform with publicly available information (https://www.worldometers.info/coronavirus/). The information on ongoing clinical trials and protocols of systematic reviews on COVID-19 is available at corresponding pages of ClinicalTrials.gov ( https://clinicaltrials.gov/ct2/results?cond=COVID-19 ) and PROSPERO registry (https://www.crd.york.ac.uk/prospero/ ).^7^ Both these registries play an important role during the current pandemic when the rush to publish results in countless redundant studies and systematic syntheses of the literature.^5^

## FILTERING MISINFORMATION

Publishing and disseminating essential updates on COVID-19 in a timely manner can potentially protect medical personnel combatting the disease on the front lines and help constrain the spread of the virus worldwide. Shortening COVID-19 manuscript turnaround times by mobilising journal editor and reviewer reserves is the most important initial step. Journal editors bear the ultimate responsibility for sieving the grain from the chaff, selecting potentially influential articles and rejecting unjustified and misleading reports that may put population health at risk.

Over the past few months, several controversial articles and social media discussion threads have surfaced in regard to the similarities between seasonal flu and COVID-19, safety of angiotensin-converting enzyme inhibitors, non-steroidal anti-inflammatory drugs (ibuprofen), methylprednisolone and other corticosteroids.^8^ Weaknesses of filtering tools at established and emerging scholarly platforms have led to the spreading of fake news via peer-reviewed journals and popular social media channels on an unprecedented scale. An example of unjustified and misleading reports relates to a widely circulated claim that patients with systemic lupus erythematosus treated with hydroxychloroquine are protected from the virus.^9^ Although strict selective approaches are important for all scholarly journals, extra efforts are particularly required at top biomedical journals which are widely read, commented on social media, and influence practice recommendations.

Displaying screened manuscripts online, ahead of print, and archiving at the PubMed Central (PMC) repository within hours or days of the acceptance is a new strategy accepted by most large publishers for creating PMC Emergency Collection. Actively disseminating links to publicly available full-texts of these articles via Twitter and other social media channels can aid public health professionals in their decision-making and save lives of patients with COVID-19.^10,11^Twitter stands out as the most popular online channel that reflects the rapidly changing situation with COVID-19 and public attention around it.^12^ Moderation of information flow on Twitter and other popular channels and analysing origins of misinformation are, however, needed to provide the end users with balanced, unbiased, and evidence-based comments and avoid unjustified deaths.^13–16^ Accordingly, involving highly skilled professionals to generate tweets and mentions on various aspects of COVID-19 is advisable during the pandemic.^17^ Arranging journal club meetings on Twitter can be also an exemplary initiative for professional societies raising their preparedness during the pandemic.^18^YouTube as one of the widely accessible free social platforms is now actively disseminating information on COVID-19 to millions of users worldwide. An analysis of 69 related video clips which were viewed by more than 257 million users revealed that government and professional videos contained factual and trustworthy information.^19^ At the same time, one-quarter of the most viewed video clips were classified as misleading.^19^ Another study of 46 YouTube links which were frequently discussing the place of hydroxychloroquine in the treatment of COVID-19 concluded that 17 (37%) consumer entries were of low quality, urging readers to watch and rely on trustworthy videos of universities and other professional institutions.^20^

As COVID-19 information flow is intensifying, several campaigns are also emerging to give a false impression that everything is under control and keep the public calm by suppressing and censoring media.^21^ The censorship obstructs the expression of concerns by social media users and authors of potentially influential scholarly articles.^22^ During the quarantine and mass restrictions, such censorship may affect all types of media and become a global issue.

## CONCLUSION

In this challenging time of the COVID-19 pandemic, all scholars should mobilize their resources to publish and disseminate evidence-based, unbiased and potentially influential scientific information that may save lives. Various established scholarly media and emerging online platforms can be employed to achieve this overarching goal. Initiatives by international organizations such as LitCovid and accessible searches to ongoing clinical trials on ClinicalTrials.gov are notable in enabling access to relevant content related to the pandemic. The involvement of professionals skilled in synthesizing evidence, distinguishing information from misinformation, and ethically disseminating units of unbiased research reports is urgently warranted. Journal editors may play a vital role by selecting potentially useful articles and actively sharing social media updates among fellows of professional societies and public at large. Wider dissemination of knowledge about the pandemic has been further enabled by numerous publishers liberalising access to COVID-19 related content.
